# Poly[(μ_3_-hydrogenphosphato)(4*H*-1,2,4-triazole-κ*N* ^1^)zinc]

**DOI:** 10.1107/S1600536812044182

**Published:** 2012-10-31

**Authors:** Hafid Aitenneite, Abdeslam El Bouari, Said Sebti, Mohamed Saadi, Lahcen El Ammari, Karim Adil

**Affiliations:** a Laboratoire de Chimie des Matériaux Solides, Faculté des Sciences Ben M’sik Casablanca, Morocco; b Laboratoire de Chimie Organique Catalyse et Environnement, Faculté des Sciences Ben M’sik Casablanca, Morocco; c Laboratoire de Chimie du Solide Appliquée, Faculté des Sciences, Université Mohammed V-Agdal, Avenue Ibn Batouta, BP 1014, Rabat, Morocco; d Laboratoire de Chimie du Solide Appliquée, Faculté des Sciences, Université Mohammed V-Agdal, Avenue Ibn Batouta, BP 1014, Rabat, Morocco.; e LUNAM Université, Université du Maine, CNRS UMR 6283, Institut des Molécules et Matériaux du Mans, Avenue Olivier Messiaen, 72085 Le Mans CEDEX 9, France

## Abstract

The asymmetric unit of the title compound, [Zn(HPO_4_)(C_2_H_3_N_3_)]_*n*_, contains one Zn^2+^ cation, one (HPO_4_)^2−^ anion and a 1,2,4 triazole ligand. The Zn^2+^ cation is coordinated in a quite regular tetra­hedral geometry by O atoms from three phosphate groups and a tertiary N atom from the triazole ring. Each phosphate anion is connected to three Zn^II^ cations, leading to a series of corrugated organic–inorganic layers parallel to the *ac* plane. The overall structure involves stacking of complex hybrid organic–inorganic layers along the *b* axis. Cohesion in the crystal is ensured by an infinite three-dimensional network of N—H⋯O and O—H⋯O hydrogen bonds between the phosphate groups and the triazole ligands.

## Related literature

For background to potential applications of similar compounds, see: Horcajada *et al.* (2012 ▶); Li *et al.* (2012 ▶); Wang *et al.* (2012 ▶); Yoon *et al.* (2012 ▶). For hybrid compounds with zinc phosphates, see: Umeyama *et al.* (2012 ▶); Horike *et al.* (2012 ▶). For phospho­nate, carboxyl­ate and azolate compounds, see: Stock & Biswas (2012 ▶). For bond-valence analysis, see: Brown & Altermatt (1985 ▶).
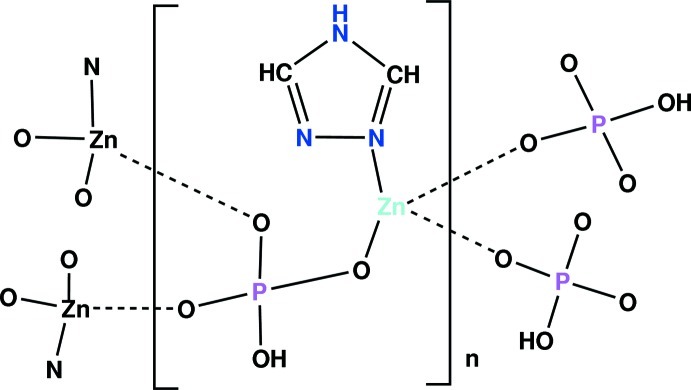


## Experimental

### 

#### Crystal data

[Zn(HPO_4_)(C_2_H_3_N_3_)]
*M* *_r_* = 230.42
Orthorhombic, 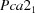

*a* = 8.5467 (13) Å
*b* = 8.4344 (12) Å
*c* = 8.9674 (13) Å
*V* = 646.43 (16) Å^3^
*Z* = 4
Mo *K*α radiation
μ = 4.01 mm^−1^
*T* = 296 K
0.24 × 0.18 × 0.12 mm

#### Data collection

Bruker X8 APEXII diffractometer
Absorption correction: multi-scan (*SADABS*; Sheldrick, 1999 ▶) *T*_min_ = 0.511, *T*_max_ = 0.638
8746 measured reflections
3318 independent reflections
3207 reflections with *I* > 2σ(*I*)
*R*_int_ = 0.029

#### Refinement

*R*[*F* ^2^ > 2σ(*F* ^2^)] = 0.021
*wR*(*F* ^2^) = 0.051
*S* = 1.04
3318 reflections
100 parameters
1 restraint
H-atom parameters constrained
Δρ_max_ = 0.46 e Å^−3^
Δρ_min_ = −1.40 e Å^−3^
Absolute structure: Flack (1983 ▶), 1184 Friedel pairs
Flack parameter: 0.020 (6)

### 

Data collection: *APEX2* (Bruker, 2005 ▶); cell refinement: *APEX2*; data reduction: *SAINT* (Bruker, 2005 ▶); program(s) used to solve structure: *SHELXS97* (Sheldrick, 2008 ▶); program(s) used to refine structure: *SHELXL97* (Sheldrick, 2008 ▶); molecular graphics: *ORTEP-3 for Windows* (Farrugia, 1997 ▶) and *DIAMOND* (Brandenburg, 2006 ▶); software used to prepare material for publication: *PLATON* (Spek, 2009 ▶) and *publCIF* (Westrip, 2010 ▶).

## Supplementary Material

Crystal structure: contains datablock(s) I, global. DOI: 10.1107/S1600536812044182/sj5276sup1.cif

Structure factors: contains datablock(s) I. DOI: 10.1107/S1600536812044182/sj5276Isup2.hkl

Additional supplementary materials:  crystallographic information; 3D view; checkCIF report

## Figures and Tables

**Table 1:** Hydrogen-bond geometry (Å, °)

*D*—H⋯*A*	*D*—H	H⋯*A*	*D*⋯*A*	*D*—H⋯*A*
N3—H3⋯O2^i^	0.86	1.99	2.8427 (15)	175
O4—H4⋯O1^ii^	0.82	1.80	2.5978 (12)	164

Symmetry codes: (i) 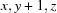; (ii) 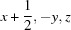.

**Fig. 1.**
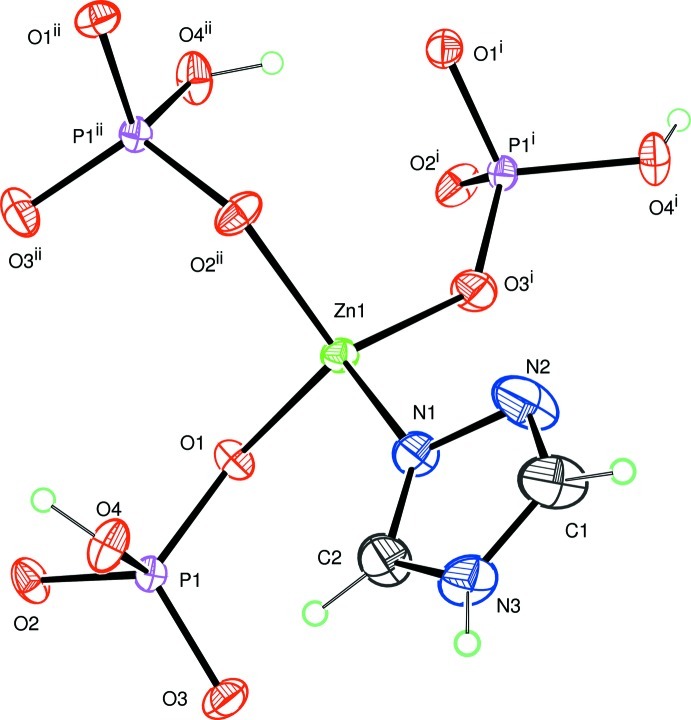
A view of the structure of the title compound showing the coordination environment of the Zn and P atoms. Displacement ellipsoids are drawn at the 50% probability level. Symmetry codes:(i) -x + 3/2, y, z + 1/2; (ii) -x + 2, -y, z + 1/2; (iii) -x + 2, -y, z - 1/2; (iv) -x + 3/2, y, z - 1/2.

**Fig. 2.**
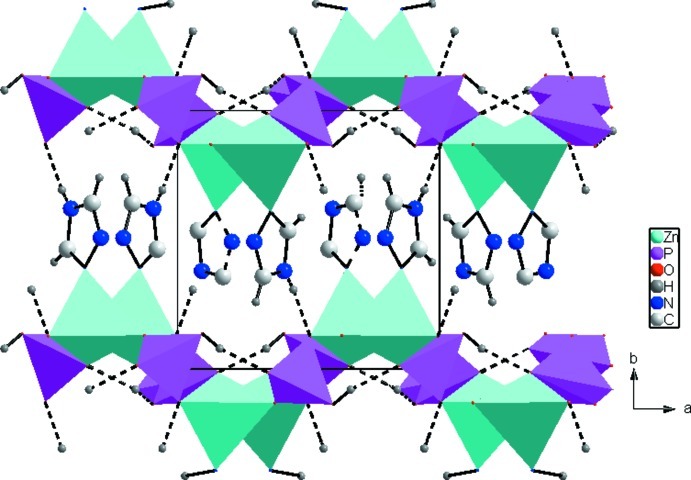
A three dimensional view of the (C2H3N3)ZnHPO4 framework structure showing a stacking of the inorganic and organic layers. The PO4 tetrahedron is shown in pink and the ZnO3N tetrahedron is blue green. Hydrogen bonds are drawn as dashed lines.

## supplementary crystallographic information

### Comment

Research into organic-inorganic hybrid materials has been an active area in recent years because of their potential applications in catalysis (Yoon *et al.*, 2012), drug-delivery (Horcajada *et al.*, 2012), enantio-selective separation (Li *et al.*, 2012) and non-linear optical materials (Wang *et al.*, 2012). A variety of new solids have been dicovered with fascinating structural architectures ranging from clusters, chains and layers to porous frameworks using phosphonates, carboxylates and/or azolates (Stock & Biswas, 2012). In this paper, we report the hydrothermal synthesis and crystal structure of a new inorganic–organic hybrid compound based on a zinc cation coordinated by three hydrogenphosphate anions and a 1,2,4-triazole ligand.

The three-dimensional structure of [(TAZ)Zn(HPO_4_)]n (HTAZ = 1,2,4 triazole) consists of infinite complex zinc-hydrogenphosphate layers. Each zinc cation is tetrahedrally coordinated by three O atoms belonging to three crystallographic equivalent phosphate groups and one azote from triazole ring (Fig.1). Zn–O distances range from 1.9172 (9) to 1.9635 (9) Å, and the Zn1–N1 distance is 1.988 (1) Å (see Table 1). The PO_4_ tetrahedron is reasonably regular with the P—O distances and O—P—O angles varying between 1.5031 (9) and 1.5730 (9) Å and 104.95 (6)° and 113.91 (6)° respectively. These values are in a good agreement with those typically observed in other phosphate based compounds (Umeyama *et al.*, 2012; Horike, *et al.*, 2012).

A three dimensional view of the crystal structure of the title compound is displayed on Fig.2. The structure can be described as the stacking of corrugated inorganic-organic layers parallel to (010) resulting from the connexion of vertex of PO_4_ groups with ZnO_3_N tetrahedra (Fig.2).

Bond valence sum calculations (Brown & Altermatt, 1985) for Zn^2+^ and P^5+^ ions are as expected, *viz*. 2.05 and 5.02 valence units, respectively. The values of the bond valence sums calculated for the oxygen atoms show low values for O4 when the contribution of H atom is not considered (*i.e.* 1.13 valence units). Hence this O atom is associated with a proton and is involved in O4—H4···O1 hydrogen bonding. The crystal structure cohesion is ensured by an infinite three-dimensional network of N3–H3···O2 and O4–H4···O1 hydrogen bonds between the phosphate groups and the triazole ligands (Table 1 and Fig.2).

### Experimental

All chemicals purchased were of reagent grade and were used without further purification. The title compound was synthesized in a hydrothermal system. A mixture of H_3_PO_4_ 85% (0.25 ml), zinc (II) nitrate hexahydrate Zn(NO_3_)_2_.6H_2_O (0.189 g), 1,2,4-triazole (0.138 g) and water (5 ml) was placed in a Parr acid digestion bomb and heated at 393 K for 48 h. The reaction vessel was allowed to cool to room temperature. Colourless crystals of (C_2_H_3_N_3_)ZnHPO_4_ were filtered off, washed with distilled water, dried in a desiccator at room temperature and manually selected for the structural determination and other characterization. The results of elemental analysis of crystals are: Zn, 28.62; P, 13.50; O, 28.02; N, 18.40; C, 10.52 and H, 0.88%.

### Refinement

The highest peak and the deepest hole in the final Fourier map are at 0.92 Å and 0.72 Å, from N1 and Zn1 respectively. H atoms were located in a difference map and treated as riding with C—H = 0.93 Å, N–H = 0.86 Å and O–H = 0.82 Å with *U*_iso_(H) = 1.2 *U*_eq_ (aromatic) and *U*_iso_(H) = 1.5 *U*_eq_ (hydroxide). The space group is not centrosymmetric and the polar axis restraint is generated automatically by the *SHELXL* program. The 1184 Friedel opposite reflections were not merged.

### Crystal data

**Table:**

[Zn(HPO_4_)(C_2_H_3_N_3_)]	*F*(000) = 456
*M**_r_* = 230.42	*D*_x_ = 2.368 Mg m^−^^3^
Orthorhombic, *P**c**a*2_1_	Mo *K*α radiation, λ = 0.71073 Å
Hall symbol: P 2c -2ac	Cell parameters from 3318 reflections
*a* = 8.5467 (13) Å	θ = 4.1–40.2°
*b* = 8.4344 (12) Å	µ = 4.01 mm^−^^1^
*c* = 8.9674 (13) Å	*T* = 296 K
*V* = 646.43 (16) Å^3^	Block, colourless
*Z* = 4	0.24 × 0.18 × 0.12 mm

### Data collection

**Table:**

Bruker X8 APEXII diffractometer	3318 independent reflections
Radiation source: fine-focus sealed tube	3207 reflections with *I* > 2σ(*I*)
Graphite monochromator	*R*_int_ = 0.029
φ and ω scans	θ_max_ = 40.2°, θ_min_ = 4.1°
Absorption correction: multi-scan (*SADABS*; Sheldrick, 1999)	*h* = −14→15
*T*_min_ = 0.511, *T*_max_ = 0.638	*k* = −13→15
8746 measured reflections	*l* = −16→14

### Refinement

**Table:**

Refinement on *F*^2^	Secondary atom site location: difference Fourier map
Least-squares matrix: full	Hydrogen site location: difference Fourier map
*R*[*F*^2^ > 2σ(*F*^2^)] = 0.021	H-atom parameters constrained
*wR*(*F*^2^) = 0.051	*w* = 1/[σ^2^(*F*_o_^2^) + (0.0181*P*)^2^] where *P* = (*F*_o_^2^ + 2*F*_c_^2^)/3
*S* = 1.04	(Δ/σ)_max_ = 0.002
3318 reflections	Δρ_max_ = 0.46 e Å^−^^3^
100 parameters	Δρ_min_ = −1.40 e Å^−^^3^
1 restraint	Absolute structure: Flack (1983), 1184 Friedel pairs
Primary atom site location: structure-invariant direct methods	Flack parameter: 0.020 (6)

### Special details

**Table:**

Geometry. All s.u.'s (except the s.u. in the dihedral angle between two l.s. planes) are estimated using the full covariance matrix. The cell s.u.'s are taken into account individually in the estimation of s.u.'s in distances, angles and torsion angles; correlations between s.u.'s in cell parameters are only used when they are defined by crystal symmetry. An approximate (isotropic) treatment of cell s.u.'s is used for estimating s.u.'s involving l.s. planes.
Refinement. Refinement of *F*^2^ against all reflections. The weighted *R*-factor *wR* and goodness of fit *S* are based on *F*^2^, conventional *R*-factors *R* are based on *F*, with *F* set to zero for negative *F*^2^. The threshold expression of *F*^2^ > 2σ(*F*^2^) is used only for calculating *R*-factors(gt) *etc*. and is not relevant to the choice of reflections for refinement. *R*-factors based on *F*^2^ are statistically about twice as large as those based on *F*, and *R*- factors based on all data will be even larger.

### Fractional atomic coordinates and isotropic or equivalent isotropic displacement parameters (Å^2^)

**Table:**

	*x*	*y*	*z*	*U*_iso_*/*U*_eq_	
Zn1	0.832301 (12)	0.167666 (13)	0.51177 (2)	0.01245 (3)	
P1	0.94990 (3)	0.03491 (3)	0.21240 (3)	0.01101 (4)	
O1	0.84382 (9)	0.01275 (12)	0.34886 (10)	0.01568 (14)	
O2	1.00560 (10)	−0.12805 (10)	0.16133 (10)	0.01765 (14)	
O3	0.87260 (11)	0.13060 (13)	0.09170 (11)	0.02070 (16)	
O4	1.09420 (9)	0.13727 (12)	0.26275 (11)	0.01867 (15)	
H4	1.1612	0.0792	0.2988	0.028*	
N1	0.85494 (13)	0.39236 (14)	0.44809 (12)	0.01976 (17)	
N2	0.7928 (2)	0.50750 (18)	0.54065 (17)	0.0380 (3)	
N3	0.90713 (16)	0.62060 (15)	0.35052 (15)	0.0269 (2)	
H3	0.9420	0.6926	0.2913	0.032*	
C1	0.8267 (2)	0.6419 (2)	0.4777 (2)	0.0356 (4)	
H1	0.7988	0.7405	0.5158	0.043*	
C2	0.9214 (2)	0.46424 (18)	0.33568 (19)	0.0295 (3)	
H2	0.9715	0.4136	0.2568	0.035*	

### Atomic displacement parameters (Å^2^)

**Table:**

	*U*^11^	*U*^22^	*U*^33^	*U*^12^	*U*^13^	*U*^23^
Zn1	0.01506 (5)	0.00996 (5)	0.01234 (5)	−0.00078 (3)	−0.00004 (4)	0.00108 (4)
P1	0.01043 (8)	0.00993 (9)	0.01266 (9)	−0.00027 (7)	−0.00016 (8)	−0.00024 (8)
O1	0.0150 (3)	0.0176 (4)	0.0145 (3)	−0.0025 (2)	0.0026 (2)	−0.0032 (3)
O2	0.0218 (3)	0.0103 (3)	0.0209 (3)	−0.0001 (3)	0.0082 (3)	−0.0016 (3)
O3	0.0171 (3)	0.0248 (4)	0.0202 (4)	0.0019 (3)	−0.0041 (3)	0.0069 (3)
O4	0.0130 (3)	0.0132 (3)	0.0298 (4)	−0.0019 (2)	−0.0056 (3)	0.0002 (3)
N1	0.0269 (4)	0.0113 (4)	0.0212 (4)	0.0000 (3)	0.0032 (3)	0.0026 (3)
N2	0.0693 (10)	0.0169 (5)	0.0279 (7)	0.0023 (6)	0.0179 (6)	0.0004 (4)
N3	0.0365 (6)	0.0144 (4)	0.0299 (5)	−0.0042 (4)	0.0016 (5)	0.0081 (4)
C1	0.0667 (13)	0.0122 (5)	0.0279 (7)	−0.0014 (6)	0.0031 (6)	−0.0021 (5)
C2	0.0412 (7)	0.0161 (5)	0.0312 (7)	0.0034 (5)	0.0128 (5)	0.0091 (5)

### Geometric parameters (Å, º)

**Table:**

Zn1—O3^i^	1.9179 (9)	O4—H4	0.8200
Zn1—O2^ii^	1.9570 (8)	N1—C2	1.3064 (18)
Zn1—O1	1.9624 (9)	N1—N2	1.3836 (19)
Zn1—N1	1.9888 (11)	N2—C1	1.299 (2)
P1—O3	1.5031 (10)	N3—C2	1.331 (2)
P1—O2	1.5250 (9)	N3—C1	1.343 (2)
P1—O1	1.5345 (9)	N3—H3	0.8600
P1—O4	1.5717 (9)	C1—H1	0.9300
O2—Zn1^iii^	1.9570 (8)	C2—H2	0.9300
O3—Zn1^iv^	1.9179 (9)		
			
O3^i^—Zn1—O2^ii^	111.25 (4)	P1—O4—H4	109.5
O3^i^—Zn1—O1	102.44 (4)	C2—N1—N2	107.71 (12)
O2^ii^—Zn1—O1	111.16 (4)	C2—N1—Zn1	134.92 (11)
O3^i^—Zn1—N1	110.58 (5)	N2—N1—Zn1	117.34 (9)
O2^ii^—Zn1—N1	106.89 (4)	C1—N2—N1	105.42 (14)
O1—Zn1—N1	114.58 (5)	C2—N3—C1	105.33 (13)
O3—P1—O2	113.89 (6)	C2—N3—H3	127.3
O3—P1—O1	112.33 (5)	C1—N3—H3	127.3
O2—P1—O1	108.30 (5)	N2—C1—N3	111.50 (16)
O3—P1—O4	104.89 (6)	N2—C1—H1	124.3
O2—P1—O4	109.66 (5)	N3—C1—H1	124.3
O1—P1—O4	107.55 (5)	N1—C2—N3	110.04 (14)
P1—O1—Zn1	122.83 (5)	N1—C2—H2	125.0
P1—O2—Zn1^iii^	125.50 (5)	N3—C2—H2	125.0
P1—O3—Zn1^iv^	139.29 (6)		

Symmetry codes: (i) −*x*+3/2, *y*, *z*+1/2; (ii) −*x*+2, −*y*, *z*+1/2; (iii) −*x*+2, −*y*, *z*−1/2; (iv) −*x*+3/2, *y*, *z*−1/2.

### Hydrogen-bond geometry (Å, º)

**Table:**

*D*—H···*A*	*D*—H	H···*A*	*D*···*A*	*D*—H···*A*
N3—H3···O2^v^	0.86	1.99	2.8427 (15)	175
O4—H4···O1^vi^	0.82	1.80	2.5978 (12)	164

Symmetry codes: (v) *x*, *y*+1, *z*; (vi) *x*+1/2, −*y*, *z*.
